# Visfatin associated with major adverse cardiovascular events in patients with acute myocardial infarction

**DOI:** 10.1186/s12872-020-01549-3

**Published:** 2020-06-05

**Authors:** Meifan Zheng, Nan Lu, Meixia Ren, Haifeng Chen

**Affiliations:** 1 Department of Cardiology, Fujian Provincial Clinical College, Fujian Medical University, Fuzhou, 350001 Fujian China; 2 Department of Cardiology, Hainan West Central Hospital, Danzhou, 571700 Hainan China; 3 Fujian Key Laboratory of Geriatrics, Department of Geriatric Medicine, Fujian Provincial Hospital, Fujian Medical University, Fuzhou, 350001 Fujian China

**Keywords:** Acute myocardial infarction, Visfatin, Major adverse cardiovascular events

## Abstract

**Background:**

Visfatin is an adipokine that related with the inflammation in atherosclerosis and the destabilization of atherosclerotic plaque. The aim of this study was to observe the relationship between visfatin and major adverse cardiovascular events (MACEs) in acute myocardial infarction (AMI) patients.

**Methods:**

We enrolled a total of 238 patients (183 AMI and 55 control) who underwent coronary angiography. Patients with AMI were followed for an average of 19.3 months and 159 patients were finally included in the study.

**Results:**

It was observed patients with AMI had higher serum visfatin levels than controls. The total incidence of MACEs was 11.32% (18/159) in AMI patients. After calculation of the Youden index, the best cut-off value of visfatin on the curve of receiver-operating characteristic was 8.799 ng/mL for predicting the occurrence of MACEs. The occurrence of MACEs was elevated in high-visfatin group (≥8.799 ng/mL) compared with low-visfatin group (≤8.799 ng/mL). The time to MACEs was correlated with visfatin (HR = 1.235, 95%CI 1.051–1.451, *P* = 0.01) and high-visfatin group had an earlier time to MACEs and a shorter time of cumulative survival.

**Conclusions:**

Increased serum visfatin levels were observed in AMI patients, and correlated with an earlier onset and higher incidence of MACEs.

## Background

Coronary atherosclerosis is a complex, long lasting and continuously evolving inflammatory disease [1]. Acute myocardial infarction (AMI) is an acute event of coronary atherosclerosis with a process involved with multiple inflammatory factors [2]. Visfatin is a novel adipokine found in 2005 by Fukuhara et al. [3]. Studies have demonstrated serum visfatin levels correlated with the presence of inflammatory state [4]. Visfatin may induce the secretion of the pro-inflammatory cytokines and could contribute to systemic and plaque inflammation in atherosclerotic disorders through impacting macrophages [5, 6]. Visfatin was found to be abundant in foam cells of unstable atherosclerotic plaques in AMI, and relevant to the destabilization of atherosclerotic plaque [7].

Previous studies have shown that visfatin was significantly increased in coronary atherosclerosis disease (CAD), and might be a promising biomarker for the diagnosis of CAD [8–11]. However, its relationship with the occurrence of severe coronary events remains unclear. Therefore, in this study, we sought to explore the association between serum visfatin levels and major adverse cardiovascular events (MACEs) in AMI.

## Methods

### Study population

A total of 238 Chinese patients, who received coronary angiography in the department of cardiology in Fujian Provincial Hospital from January 2016 to September 2016 were recruited for this study. AMI group (*N* = 183, 62 ST-segment elevation myocardial infarction and 121 Non-ST segment elevation myocardial infarction) was defined with more than 50% stenosis in coronary arteries and positive troponin. The diagnostic criteria of AMI adopted the American College of Cardiology (ACC)/American Heart Association (AHA)/World Heart Federation (WHF)/European Society of Cardiology (ESC) guidelines established in 2012 [12]. Control group (*N* = 55) was defined with less than 50% stenosis in coronary arteries and negative troponin. The exclusion criteria as follows: valvular heart disease, myocarditis, cardiomyopathy, hypohepatia, thyroid dysfunction, end-stage chronic kidney disease, malignancy and hematological system diseases. The study complied with the Declaration of Helsinki and was approved by the ethical committee of Fujian Provincial Hospital (K2016–01-001) (supplemental materials). Each participant provided written, informed consent before enrollment.

### Patient information and laboratory examination

Patient information (such as age, gender, body mass index, hypertension, diabetes, hyperlipidemia, history of myocardial infarction and smoking) was recorded by standardized form. The cardiac biomarkers (cardiac troponin-I, N-terminal pro-brain natriuretic peptide) and thrombin index (fibrinogen, D-dimer) were detected immediately after admission. Other vein biochemical index were detected in the next morning (fasting for 8–12 h) after admission, including glucose, albumin, creatinine, triglycerides, total cholesterol, high-density lipoprotein, low-density lipoprotein, apolipoprotein-a, apolipoprotein-b. The above indicators were completed by the laboratory department of our hospital and we obtain laboratory parameter from the patients medical records at index admission.

### Visfatin detection

Blood samples were collected into EDTA-containing tubes from subjects at baseline at least 8–12 h fasting before coronary angiography and centrifuged at 700 g. Serum concentration of visfatin was determined by enzyme-linked immunosorbent assay with the EK-003-80 human visfatin kit (Phoenix Pharmaceuticals, Belmont, CA). The operation steps followed the instructions strictly.

### Coronary angiography

Standard Judkins technique was carried out in the coronary arteriography and all coronary artery stenosis was imaged from multiple projections. Two expert cardiologists who were blinded to the patients’ clinical and laboratory data reviewed the coronary angiography and evaluated the coronary atherosclerotic lesion severity independently. According to the number of stenosis which was detected ≥50% of the lumen diameter of a coronary artery, we divided it into single-vessel lesion, double vessel lesion and multi-vessel lesion.

### Follow-up

In this study, all of AMI patients were followed up after admission using a standardized protocol that included outpatient follow up, telephone contacts and hospital data. The endpoints was MACEs, including death of cardiovascular events, nonfatal myocardial infarction (re-MI), target lesion revascularization (percutaneous coronary intervention or coronary artery bypass graft) and re-admission due to advanced heart failure.

### Statistical analysis

The version 22.0 SPSS software suite was used for all statistical analyses. Continuous variables was tested for normality test. Normally distributed data was expressed as mean ± standard deviation and skewed distributions data was expressed as median with inter-quartile range. Intergroup comparisons of clinical data were performed with student’s t-test (normally distributed data) or the Mann–Whitney U test (skewed data). Multiple groups comparisons of clinical data were performed with the analysis of variance (normally distributed data) or the Kruskal-Wallis H test (skewed data). Categorical variables were presented as number (percentage) and analyzed by chi-square statistic test. Binary Logistic regression was used to analyze the factors which were correlated with the occurrence of MACEs in a stepwise backward conditional manner. Receiver-operating characteristic (ROC) curves was used to evaluated the predictive value of visfatin for the occurrence of MACEs. Cox regression analysis was used to analyze the factors which were correlated with the time to MACEs and Kaplan-Meier curves was used to estimate the survival time between high and low visfatin group with a log-rank test. For all analyses, two tailed *P* values < 0.05 were.

## Results

### Baseline characteristics

A total of 238 participants (183 AMI and 55 control group, mean age 64.40 ± 10.68 years). The baseline characteristics of participants were summarized in Table 1. Compared with control group, AMI group had more males, higher smoking history and the prevalence of hypertension, but was younger and had lower systolic blood pressure. The AMI group had significantly higher fasting blood glucose, ApoB, creatinine, fibrinogen, D-Dimer, CTnI, NT-proBNP (all *P* <  0.05) and lower albumin, HDL-C, ApoA (all *P* <  0.05) compared with control group. However, there were no statistical significant in body mass index (BMI), diabetes, hyperlipidemia, diastolic blood pressure, triglycerides, total cholesterol, LDL-C between two groups (all *P* > 0.05). Meanwhile, serum visfatin levels were significantly higher in AMI than control group [7.70 (6.57, 9.82) vs 6.74 (5.76, 7.53), *P* <  0.01].

**Table 1:** Clinical characteristics of control and AMI patients

Variables	Control (*n* = 55)	AMI (*n* = 183)	*P* value
Male (n, %)	33 (60.00%)	155 (84.70%)	< *0.001*
Age (years)	66.87 ± 9.45	63.66 ± 10.94	*0.036*
BMI (kg/m^2^)	23.48 ± 2.32	23.83 ± 2.84	*0.349*
Current smoking (n, %)	17 (30.90%)	98 (53.55%)	*0.003*
Diabetes (n, %)	8 (14.54%)	46 (25.14%)	*0.100*
Hypertention (n, %)	15 (27.27%)	96 (52.40%)	*0.001*
Hyperlipidemia (n, %)	14 (25.45%)	55 (30.05%)	*0.510*
SBP on admission (mmHg)	137.69 ± 21.78	128.69 ± 19.80	*0.004*
DBP on admission (mmHg)	74.34 ± 10.52	75.88 ± 11.85	*0.358*
Glucose (mmol/L)	5.57 ± 1.53	6.23 ± 2.04	*0.012*
Albumin (g/L)	40.87 ± 4.32	39.45 ± 4.26	< *0.001*
Triglycerides (mmol/L)	1.38 ± 0.62	1.52 ± 0.86	*0.168*
Total cholesterol (mmol/L)	4.22 ± 0.91	4.28 ± 1.00	*0.659*
HDL-C (mmol/L)	1.22 ± 0.34	1.05 ± 0.29	< *0.001*
LDL-C (mmol/L)	2.58 ± 0.79	2.77 ± 0.89	*0.133*
Apo A (g/L)	1.29 ± 0.24	1.15 ± 0.36	*0.001*
Apo B (g/L)	0.83 ± 0.23	0.92 ± 0.26	*0.010*
Creatinine (umol/L)	73.29 ± 18.83	81.58 ± 25.99	*0.010*
Fibrinogen (g/L)	3.35 ± 0.84	4.24 ± 1.23	< *0.001*
D-Dimer (mg/L)	0.3 (0.19,0.44)	0.46 (0.30,0.79)	< *0.001*
CTnI (ng/mL)	0.01 (0.00,0.01)	5.62 (1.10,28.28)	< *0.001*
NT-proBNP (pg/mL)	124 (59.00,200.00)	690 (278.00,1855.00)	< *0.001*
Visfatin (ng/mL)	6.74 (5.76,7.53)	7.70 (6.57,9.82)	< *0.001*

### Serum visfatin levels in different number of coronary lesions

There were 70 (38.25%) single vessel coronary lesions, 57 (31.15%) double vessel coronary lesions, 56 (30.60%) multiple vessel coronary lesions in AMI patient after coronary angiography (Table 2). As indicated by Mann-Whitney U test, serum visfatin levels of single, double and multiple vessel coronary lesions were significantly higher than control group (*P* <  0.05). However, Kruskal-wallis H test analysis showed that there were no statistically significant difference between the groups (*P* > 0.05).

**Table 2:** Serum visfatin levels in different number of coronary lesions

Variables	Case (n)	Visfatin (ng/mL)
control group	55	6.74 (5.76,7.53)
Single vessel coronary lesions	70	7.68 (6.37,9.65)*
Double vessel coronary lesions	57	7.67 (6.66,8.92)*
Multiple vessel coronary lesions	56	8.14 (6.63,11.60)*

* Compared with control group, *P* <0.05

### MACEs during follow-up

A total of 183 patients with AMI were followed up with the period of 16–24 months (average 19.3 months), 24 patients were excluded because of various reasons, only 159 patients received follow-up. Total 18 (11.32%) patients presented with MACEs. Of these, 7 (4.40%) suffered non-fatal re-MI, 2 (1.26%) suffered heart failure, 9 (5.65%) were dead of cardiovascular events.

A total of 159 patients were divided into MACEs group and non-MACEs group, the clinical characteristics analysis showed that MACEs group had significantly higher age, diabetes, creatinine, NT-proBNP, serum visfatin levels (all *P* <  0.05) and lower albumin, triglycerides, total cholesterol, LVEF (all *P* <  0.05) than non-MACEs group. In contrast, no significant differences were observed in gender, smoking, hypertension, hyperlipaemia, history of MI, type of AMI, BMI, systolic pressure, diastolic pressure, glucose, HDL-C, LDL-C, ApoA, ApoB, fibrinogen, D-Dimer and CTnI (Table 3).

**Table 3:** Clinical characteristics between MACEs and non-MACEs

Variables	MACEs (n = 18)	non-MACEs (*n* = 141)	*P* value
Male (n, %)	13 (72.22%)	122 (86.52%)	*0.139*
Age (years)	68.89 ± 8.99	62.23 ± 10.58	*0.008*
Current smoking (n, %)	8 (44.44%)	78 (52.48%)	*0.384*
Hypertension (n, %)	13 (72.22%)	70 (49.64%)	*0.066*
Hyperlipidemia (n, %)	3 (16.67%)	44 (31.20%)	*0.181*
Diabetes (n, %)	9 (50.00%)	32 (22.69%)	*0.019*
History of MI (n, %)	3 (16.67%)	18 (12.67%)	*0.655*
STEMI (n, %)	3 (16.67%)	50 (35.40%)	*0.093*
BMI (kg/m^2^)	24.35 ± 2.55	33.85 ± 2.98	*0.450*
SBP on admission (mmHg)	124.61 ± 17.44	128.84 ± 19.54	*0.349*
DBP on admission (mmHg)	74.05 ± 13.76	75.83 ± 11.61	*0.606*
Glucose (mmol/L)	7.06 ± 3.06	6.07 ± 1.76	*0.192*
Albumin (g/L)	37.44 ± 4.30	39.84 ± 4.19	*0.037*
Triglycerides (mmol/L)	1.21 ± 0.49	1.54 ± 0.80	*0.021*
Total cholesterol (mmol/L)	3.77 ± 0.97	4.35 ± 0.99	*0.030*
HDL-C (mmol/L)	0.92 ± 0.26	1.05 ± 0.27	*0.068*
LDL-C (mmol/L)	2.45 ± 0.78	2.84 ± 0.88	*0.061*
Apo A (g/L)	1.19 ± 0.95	1.14 ± 0.22	*0.535*
Apo B (g/L)	0.87 ± 0.23	0.95 ± 0.25	*0.146*
Creatinine (umol/L)	101.67 ± 46.63	79.88 ± 22.59	*0.001*
Fibrinogen (g/L)	4.04 ± 0.86	4.24 ± 1.32	*0.634*
D-Dimer (mg/L)	0.52 (0.36,1.48)	0.42 (0.25,0.79)	*0.084*
CTnI (ng/mL)	8.44 (1.09,40.35)	4.77 (1.02,28.74)	*0.518*
NT-proBNP (pg/mL)	1751 (372.00,9262.50)	575 (248.00,1423.50)	*0.025*
LVEF (%)	0.51 ± 0.02	0.57 ± 0.01	*0.006*
Visfatin (ng/mL)	9.89 (8.66,12.80)	7.53 (6.53,8.94)	*0.001*

### Correlation of visfatin with MACEs

Binary logistic regression was used to analyze the predictors of MACEs, after adjusting for gender and age, visfatin, total cholesterol, LDL-C and diabetes were correlated with the occurrence of MACEs in AMI patients. (Table 4).

**Table 4:** Risk factors of MACEs in AMI

Variables	OR	95%CI	*P* Value
Gender	0.225	0.045–1.119	*0.068*
Age	1.025	0.932–1.127	*0.614*
Visfatin	1.278	1.037–1.574	*0.021*
Total cholesterol	0.028	0.001–0.521	*0.017*
LDL-C	2.990	1.142–7.825	*0.041*
Diabetes	3.766	1.053–7.473	*0.041*

**Fig. 1:**
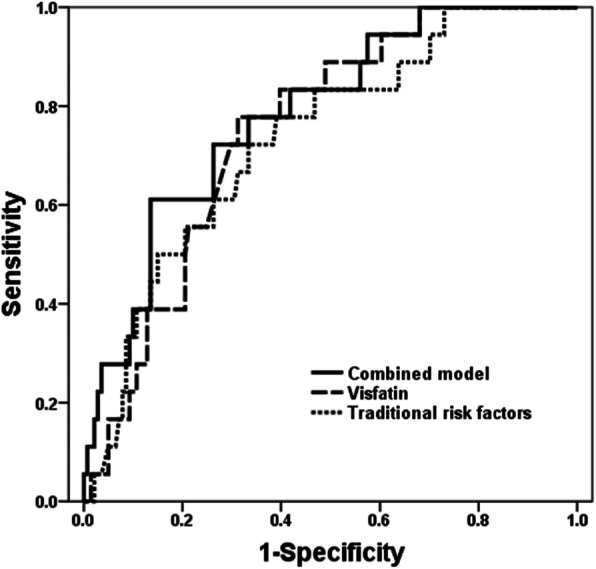
Receiver-operating characteristic curves of visfatin, traditional risk factors, and combined model for predicting MACEs

Contribution of visfatin in predicting the occurrence of MACEs was assessed by ROC curves which showed that visfatin had a 0.751 area under curve (AUC) (95%CI 0.652–0.849, *P* = 0.010) with a 78.8% sensitivity and 68.8% specificity. The traditional risk factors, including smoking, hypertension, diabetes, hyperlipidemia and BMI had a 0.733 AUC (95%CI 0.619–0.847, *P* = 0.010) with a 72.2% sensitivity and 66.7% specificity. While, a combined model consisting of visfatin and traditional risk factors (smoking, hypertension, diabetes, hyperlipidemia, BMI) showed a 0.783 AUC with a 72.2% sensitivity and 73.8% specificity (Fig. 1).

After calculation of the Youden index, the best cut-off value of visfatin on the ROC curves was 8.799 ng/mL for predicting the occurrence of MACEs. Patients were divided into high-visfatin group (serum visfatin levels ≥8.799 ng/mL) and low-visfatin group (serum visfatin levels ≤8.799 ng/mL) based on the threshold. By chi-square test, we found that the occurrence of MACEs was elevated in high-visfatin group compared with low-visfatin group, and the occurrence of non-fatal re-MI was elevated in high-visfatin group (*P* = 0.049, Table 5).

**Table 5:** MACEs of high-visfatin group and low-visfatin group

Variables(n,%)	Visfatin≥8.799 ng/ml (*n* = 58)	Visfatin≤8.799 ng/ml (*n* = 101)	*P* value
MACEs	11 (18.96%)	7 (6.93%)	*0.021*
non-fatal re-MI	5 (8.62%)	2 (1.96%)	*0.049*
heart failure	1 (1.72%)	1 (0.99%)	*0.698*
death of cardiovascular events	5 (8.62%)	4 (3.96%)	*0.227*

### Survival curve analysis of AMI patients in follow-up

**Fig. 2:**
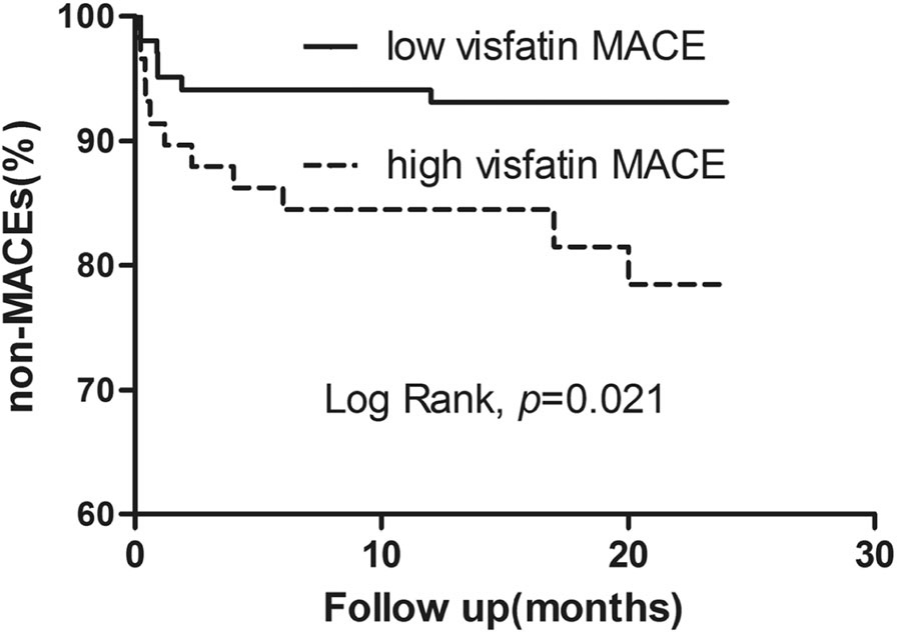
The cumulative event-free survival analysis of high-visfatin group and low-visfatin group

The univariate COX proportional hazards regression analysis showed that age, diabetes, glucose, albumin, total cholesterol, creatinine, D-Dimer, LVEF and visfatin were associated with the time to MACEs. While, the multivariate Cox proportional hazards regression analysis showed that after adjusting for gender and age, only visfatin correlated with the time to MACEs (Table 6). Further, the Kaplan-Meier cuvers demonstrated that the time to MACEs was earlier in high-visfatin group compared with low-visfatin group, and the cumulative survival time was shorter (*P* = 0.021, Fig. 2).

**Table 6:** Univariate and Multivariate Cox Regression of MACEs

Variables	Univariate COX	*P* value	Multivariate COX	*P* value
	HR (95%CI)		HR (95%CI)	
Age	1.063 (1.012,1.117)	*0.014*	1.011 (0.952,1.075)	*0.715*
Gender	0.435 (0.155,1.222)	*0.114*	2.447 (0.712,8.407)	*0.155*
Diabetes	3.061 (1.215,7.712)	*0.018*	0.466 (0.139,1.566)	*0.217*
Glucose	1.208 (1.021,1.430)	*0.028*	1.152 (0.953,1.392)	*0.143*
Albumin	0.885 (0.792,0.988)	*0.029*	1.020 (0.870,1.196)	*0.803*
Total cholesterol	0.576 (0.352,0.942)	*0.028*	0.595 (0.336,1.054)	*0.075*
Creatinine	1.018 (1.007,1.029)	*0.001*	1.010 (0.997,1.022)	*0.124*
D-Dimer	1.531 (1.178,1.990)	*0.001*	1.333 (0.955,1.862)	*0.091*
LVEF	0.014 (0.001,0.274)	*0.005*	0.011 (0.000,4.913)	*0.148*
Visfatin	1.226 (1.076,1.398)	*0.002*	1.235 (1.051,1.451)	*0.010*

## Discussion

In the present study, we found that serum visfatin level was elevated in AMI patients and contributed to MACEs incidence. Visfatin, total cholesterol, LDL-C and diabetes were correlated with the occurrence of MACEs in AMI patients. A combined model consisting of visfatin and traditional risk factors showd a higher specificity in predicting MACEs. The occurrence of MACEs was elevated in high-visfatin group, especially in non-fatal re-MI. Visfatin was correlated with the onset to MACEs and high serum visfatin level was associated with an earlier onset of MACEs.

Visfatin (also referred as Pre-B cell colony enhancing factor and nicotinamide phosphoribosyltransferase) is a member of adipokine with a molecular weight about 52kD [13]. Studies have demonstrated that visfatin could promote insulin and pro-inflammatory cytokines secretions [5, 14], and it is a pleiotropic protein implicated in the pathophysiology of obesity, metabolic disease, diabetes and cancer [15–18]. Dahl et al. firstly reported that visfatin was markedly enhanced in symptomatic carotid atherosclerotic plaques [19]. Subsequently, various studies provided that visfatin was closely related with cardiovascular disease [20–26]. Horbal et al. showd that the odds of severe hypertension were in accordance with the levels of visfatin [21]. Bobbert et al. discovered high visfatin in nonischemic dilated congestive cardiomyopathy (DCM) patients was associated with a favorable outcome and improvement in functional status [22]. In addition, serum visfatin level was observed to be significantly higher in premature coronary artery disease and coronary slow flow (CSF) [23, 24]. Notably, an augmented level of visfatin in STEMI was positively correlated with the number of coronary lesions [10, 25]. We also found an increase of serum visfatin levelin AMI patients. However, there was no statistically significant difference in the number of coronary lesions, possibly because of the limited number of subjects in our study.

In this study, an average of 19.3 months of follow-up was conducted for 159 patients with AMI. A total of 18 (11.32%) patients had MACEs in follow-up, among those, 7 (4.40%) suffered non-fatal re-MI, 2 (1.26%) suffered heart failure, 9 (5.65%) were dead of cardiovascular events. Binary logistic analysis showed that visfatin, total cholesterol, LDL-C and diabetes were all associated with the occurrence of MACEs.

We applied ROC curves to evaluate the contribution of traditional risk factors (smoking, hypertension, diabetes, hyperlipidemia and body mass index) and visfatin in predicting MACEs. We discovered that visfatin and traditional risk factors had a specificity of 68.8 and 66.7% in predicting MACEs,respectively, while a combined model consisting of all factors showed a 73.8% specificity. The combined model greatly improved the predictive of MACEs and clinical practical value. Our data showed the occurrence of MACEs was elevated in high-visfatin group, especially in non-fatal re-MI.

Multivariate Cox proportional hazards regression analysis suggested that visfatin was correlated with the onset to MACEs. Kaplan-Meier curves demonstrated that the onset to MACEs was earlier in high-visfatin group compared with low-visfatin group, and the cumulative survival time was shorter. Although the mechanisms of visfatin in coronary atherosclerotic heart disease remain unclear, previous researchers had shown the increased serum visfatin level after drug-eluting stents (DES) placement was independently associated with in-stent restenosis (ISR) [26]. Visfatin could promote the production of interleukin (IL)-6 and intercellular adhesion molecule-1 (ICAM-1) and regulate nuclear factor (NF)-kappaB, which resulted in the apoptosis of endothelial progenitor cells (EPCs) [5]. Visfatin could also increase the expression of metal matrix proteinase (MMP)-8 in macrophages, promote collagen degradation and the plaques vulnerability index [27]. Visfatin was indicated to be abundant in foam cells within unstable atherosclerotic plaques [7]. Therefore, we presume visfatin may contribute to the MACEs by regulating the inflammation, apoptosis and collagen degradation, and we will take further clinical research and basic experimental to investigate the possible mechanism behind.

There were several limitations in the present study. First, the subjects recruited were hospitalized in single centre, lacking regional and ethnic comparisons. Second, the sample size was limited and the follow-up time was relatively short. Third, lacking of dynamic monitoring of visfatin levels during the follow-up period.

## Conclusions

Our findings support previous observations of serum visfain level was elevated in acute myocardial infarction patients. In addition, our study demonstrated that visfatin was correlated with an earlier onset and higher incidence of MACEs.

## Supplementary information

**Additional file 1.** Ethics approval.

## Data Availability

The datasets generated and analysed during the current study are not publicly available due to protection of individual privacy, but are available from the corresponding author on reasonable request.

## Abbreviations

AMI: Acute myocardial infarction
Apo A: Apolipoprotein-a
Apo B: Apolipoprotein-b
AUC: Area under curve
BMI: Body mass index
CAD: Coronary atherosclerosis disease
CTnI: Cardiac troponin-I
HDL-C: High-density lipoprotein
LDL-C: Low-density lipoprotein
LVEF: Left ventricular ejection fraction
MACEs: Major adverse cardiovascular events
MI: Acute myocardial infarction
NT-proBNP: N-terminal pro-brain natriuretic peptide
ROC: Receiver-operating characteristic
