# Supplementary material for: Visfatin associated with major adverse cardiovascular events in patients with acute myocardial infarction
Source: BMC Cardiovasc Disord. 2020 Jun 5;20:271. doi: 10.1186/s12872-020-01549-3 (PMC7275306; doi:10.1186/s12872-020-01549-3)

**Fujian Provincal Hospital Clinical Ethics**

**Research Committee**

Ethics Research Number(K2016-01-001)

| Review  project | Project Name | | Serum level of visfatin and FGF21 in patients with coronary artery disease | | |
| --- | --- | --- | --- | --- | --- |
|  | Funding | | National Natural Science Foundation of China (81470021) and the Key Project of Cultivating Young Talent in Fujian provincial health and family planning commission (2016-ZQN-8). | | |
|  |  |  |  |  |  |
|  | Project Numeber | | / | Time periods | Jan 2016------Jan 2019 |
|  | Department | | Cardiology | Project leader | Hai-feng Chen |
|  | Position | | Chief | Contact number | +86 13705930769 |
| Documents | 1. Clinical research plan 2. Informed consent 3. Application of ethical review | | | | |
| Methods | | Quick review | | | |
| Results | According to the principles of Ethics review method involving human biomedical research of the Ministry of Health (2007), the Drug clinical trial management standard of cFDA (2003), the Medical device clinical trial regulation (2004), the Helsinki declaration (2008) and the International ethics guide for human biomedical research (2002) , this clinical research and documents had be appoved by the ethics committee .  Whether or not this research passed the ethical committee's review ☑Yes □ No  Review frequency for this research □ Six months ☑ One year | | | | |
| Ethics research committee: | | | | | |

Address: Fujian Provincial hospital, 134 Dongjie, Fuzhou, Fujian 350001, P.R. China Tel: 0591-88216141


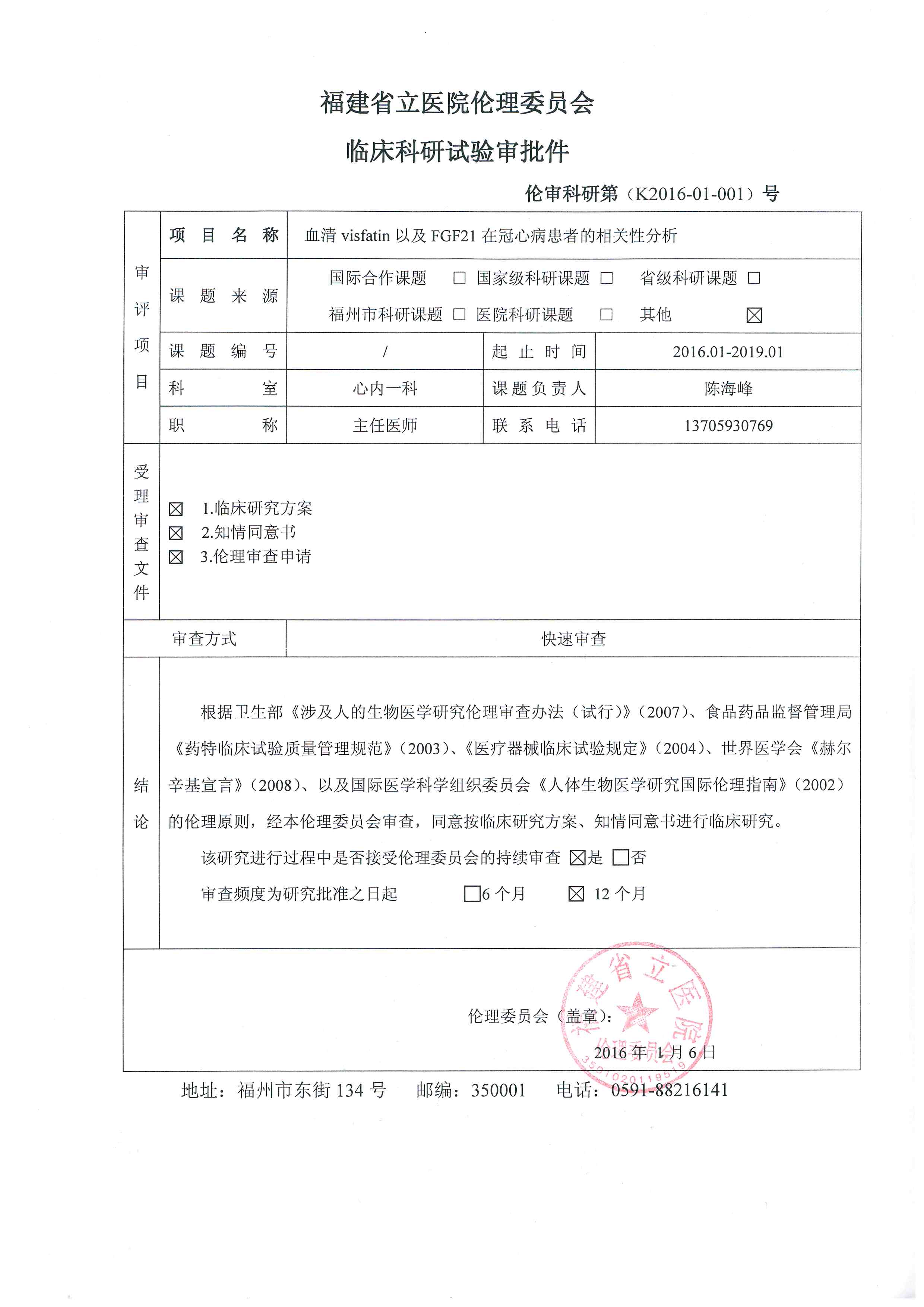

Supplement: Supplementary file 1 — Additional file 1. Ethics approval. [file 12872_2020_1549_MOESM1_ESM.doc]
